# Cementless total hip arthroplasty in patients with severely dysplastic hips and a previous Schanz osteotomy of the femur: Techniques, pitfalls, and long-term outcome

**DOI:** 10.3109/17453670902967273

**Published:** 2009-06-01

**Authors:** Antti Eskelinen, Ville Remes, Pekka Ylinen, Ilkka Helenius, Kaj Tallroth, Timo Paavilainen

**Affiliations:** ^1^ORTON Orthopedic Hospital, Invalid FoundationHelsinkiFinland; ^2^Department of Orthopedics and Traumatology, Helsinki University Central HospitalHelsinkiFinland; ^3^Coxa Hospital for Joint ReplacementTampereFinland; ^4^Hospital for Children and Adolescents, Helsinki University Central HospitalHelsinkiFinland

## Abstract

**Background and purpose** Historically, a Schanz osteotomy of the femur has been used to reduce limp in patients with severely dysplastic hips. In such hips, total hip arthroplasty is a technically demanding operation. We report the long-term results of cementless total hip arthroplasty in a group of patients who had all undergone a Schanz osteotomy earlier.

**Patients and methods** From 1988 through 1995, 68 total hip replacements were performed in 59 consecutive patients previously treated with a Schanz osteotomy. With the cup placed at the level of the true acetabulum, a shortening osteotomy of the proximal part of the femur and distal advancement of the greater trochanter were performed in 56 hips. At a mean of 13 (9–18) years postoperatively, we evaluated these patients clinically and radiographically.

**Results** The mean Harris hip score had increased from 51 points preoperatively to 93 points. Trendelenburg sign was negative and there was good or slightly reduced abduction strength in 23 of 25 hips that had not been revised. There were 12 perioperative complications. Only 1 cementless press-fit porous-coated cup was revised for aseptic loosening. However, the 12-year survival rate of these cups was only 64%, as 18 cups underwent revision for excessive wear of the polyethylene liner and/or osteolysis. 6 CDH femoral components had to be revised due to technical errors.

**Interpretation** Our results suggest that cementless total hip arthroplasty combined with a shortening osteotomy of the femur and distal advancement of the greater trochanter can be recommended for most patients with a previous Schanz osteotomy of the femur. Because of the high incidence of liner wear and osteolysÍs of modular cementless cups in this series, nowadays we use hard-on-hard articulations in these patients.

## Introduction

In 1922, Schanz described the technique of subtrochanteric osteotomy of the femur for young adults with high dislocation of the hip joint (Schanz 1922). The purpose of the procedure was to reduce severe limp and to improve abduction and flexion. Early attempts to reconstruct the hips of adults who had high congenital dislocation were unsuccessful owing to the associated anatomical abnormalities (Hartofilakidis et al. 1996), and some authors considered high hip dislocation to be a contraindication for total hip replacement (Charnley and Feagin 1973, Eftekhar 1978). After a Schanz osteotomy, the anatomy is even more altered and variable on both the femoral and the pelvic sides (Paavilainen et al. 1990, 1993, Paavilainen 1997). Identification and preparation of the true acetabulum, preparation of the femoral canal, and stable reduction of the components pose several technical problems.

There have only been 5 previous reports of total hip arthroplasty including patients who have previously undergone a Schanz osteotomy (Paavilainen et al. 1990, 1993, Perka et al. 2000, Sener et al. 2002, Eskelinen et al. 2006). In these studies, patients with a previous Schanz osteotomy represented only a small subgroup of the study population.

We describe the technique and the pitfalls required for successful conversion, and review the long-term clinical and radiographic outcomes of cementless total hip arthroplasty in 59 patients (68 hips) previously treated with a Schanz osteotomy.

## Patients and methods

Between January 1988 and December 1995, 75 primary total hip arthroplasties were performed at our hospital in 65 consecutive patients who had previously undergone a Schanz osteotomy of the femur. The operations were all performed by, or under the direct supervision of, 9 senior orthopedic surgeons, and two-thirds of them were performed by the senior author (TP). The indications for arthroplasty were severe pain and/or considerable difficulty in walking and performing daily activities. 4 patients died (5 hips) at a mean of 9 years after the operation, from causes unrelated to the procedure. 59 of the remaining 61 patients agreed to participate in this follow-up study (68 hips) (Tables 1 and 2). 2 declined, giving lack of interest as their reason for not participating; both of them had undergone revision of the femoral component. At a mean of 13 (9–18) years after the primary arthroplasty, we performed a physical examination on 58 patients (67 hips) and radiographic examination of all 59 patients (68 hips): 1 patient was not available for physical examination (1 hip) because of a medical condition unrelated to her hip disease; this patient was contacted by telephone, and new radiographs were taken at her local hospital and sent to us for further analysis.

**Table 1.** Classification of the hips according to Eftekhar (1978) and Hartofilakidis et al. (1996)

		Eftekhar classification (n **^b^**)			
Hip disease **^a^**	n **^b^**	A	B	C	D
Congenital hip disease (n = 63)					
Dysplasia	4	4			
Low dislocation **^c^**	9		9		
High dislocation	51			18	33
Tuberculosis (n = 3) **^c^**					
Dysplasia	1	1			
High dislocation	2			1	1
Coxa vara (n = 1) **^c^**					
Dysplasia	1	1			

**^a^** Classification of dysplasia according to Hartofilakidis et al. (1996).

**^b^** n: number of hips.

**^c^** Hips with previous tuberculous coxarthritis or coxa vara were classified with the same methods.

**Table 2.** Clinical characteristics at the time of primary arthroplasty

Male/female (no. of patients)	5 / 54
Median age (range) at time of operation (yr)	50 (29–69)
Median height (range), m	1.55 (1.40–1.73)
Median weight (range), kg	63 (44–87)
No. of hips Trendelenburg +/−	52 / 16

55 patients (64 hips) had congenital hip disease, which was graded as high dislocation in 53 hips.

The short-term results for 18 patients (20 hips) with a previous Schanz osteotomy and the long-term results for 28 patients (34 hips) with high congenital hip dislocation have been reported previously (Paavilainen et al. 1993, Eskelinen et al. 2006).

### Operative technique

**Figure 1.**
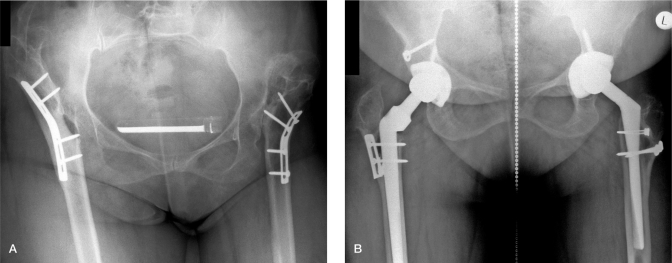
A 54-year-old woman who had high congenital dislocation of both hips. At the age of 17, she had undergone a bilateral high-seated Schanz osteotomy to reduce limp. A. Preoperatively. B. 9 years after a cementless total hip arthroplasty of the left hip and 8 years after a similar procedure on the left hip. Both hips underwent femoral shortening and advancement of the greater trochanter. The leg-length discrepancy was 1 cm (the left side being shorter). There were no radiographic signs of loosening of the components and no signs of polyethylene wear.

**Figure 2.**
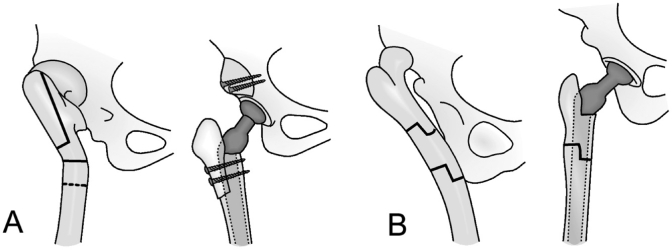
Osteotomies used for various deformities of the femur. The femoral shaft is usually transected distal to the lesser trochanter, as shown by the transverse solid line in (A). A dotted line demonstrates the most distal possible level of the osteotomy. A. Proximal shortening osteotomy with distal advancement of the greater trochanter (vertical solid line) in hips with a previous proximal Schanz osteotomy. B. Segmental shortening with angular correction for hips with a previous, more distal Schanz osteotomy. Copyright for the illustrations in this figure is owned by The Journal of Bone and Joint Surgery, Inc. (published in Eskelinen et al. Cementless total hip arthroplasty in patients with high congenital hip dislocation, J Bone Joint Surg Am. 2006; 88: 80-91). Reproduced with permission.

*Preparation of the femur.* The appropriate operative procedure was selected according to the level of the Schanz osteotomy, and was performed by one of the two methods described in detail elsewhere (Paavilainen et al. 1990, 1993, Paavilainen 1997, Eskelinen et al. 2006). Only 4 hips could be replaced without femoral shortening osteotomy. 56 hips were replaced after having performed a shortening osteotomy of the proximal part of the femur with transposition of the greater trochanter (Figures 1 and 2). In 8 hips, the Schanz osteotomy had been performed so low that the first method (shortening osteotomy performed at the level of the Schanz angle) would have resulted in inadequate limb-length correction. Thus, a metaphyseal segmental shortening osteotomy with angular correction was performed at the level of the Schanz angle, and a step method was used to stabilize the osteotomy against rotation (Paavilainen et al. 1990). The stem was used as an intramedullary nail to stabilize the osteotomy site (Figure 2). This operation was performed through the modified anterolateral approach described by Hardinge (1982), which provides better access for the corrective osteotomy.

*Preparation of the acetabulum.* The small rudimentary acetabulum that is usually seen in untreated high dislocation was often deformed and remodeled by the articulation between the Schanz angle and the pelvic wall. The new acetabulum was created at the anatomic level between the pubic and ischial bones using techniques that have been described in detail elsewhere (Paavilainen et al. 1990, 1993, Paavilainen 1997, Eske-linen et al. 2006).

The median duration of the operation was 3.5 (2–5.5) hours. The median perioperative blood loss was 2 (0.5–10) L. The patient with perioperative blood loss of 10 L underwent THA with a metaphyseal segmental shortening osteotomy; despite the technically demanding operation and massive blood loss, there were no perioperative complications. In bilateral cases, the median interval between operations was 10 (7–13) months.

A cementless CDH stem (Biomet, Warsaw, IN) combined with either a smooth-threaded or a press-fit porous-coated cementless cup was used in most of the operations (Table 3). The choice of the stem depended on the anatomy of the proximal part of the femur beyond the resection level. The median outer diameter of the acetabular components was 46 (40–56) mm. The outer diameter of the cup was ≤ 44 mm in 15 of the 68 hips, and for these only 22-mm heads were available. In the larger cups, we used 28-mm heads.

**Table 3.** Prosthetic components

Implant	n	Material	Porous coating	Design	Dates	Liner
Stem						
Lord Madréporique	1	Cobalt-chromium	Full	Straight, intramedullary rod	1988	–
Biomet CDH (Collared)	46	Titanium alloy	Proximal	Straight, collared	1988–1995	–
Biomet CDH (Collarless)	17	Titanium alloy	Proximal	Straight, collarless	1993–1995	–
Biomet Head-Neck	2	Titanium alloy	Proximal	Straight, tapered, box-like collar	1988–1990	–
Biomet Bi-Metric	2	Titanium alloy	Proximal	Straight, tapered	1994–1995	–
Cup						
Biomet T-Tap	18	Titanium alloy	No **^a^**	Conical	1988–1989	HexLocTM
Biomet Universal	24	Titanium alloy	Yes	Hemispherical	1989–1994	HexLocTM
Biomet Mallory	18	Titanium alloy	Yes	Hemispherical, with fins	1989–1992	HexLocTM
Biomet Vision	8	Titanium alloy	Yes	Hemispherical	1995	RingLocTM

**^a^** Smooth threaded

*Clinical evaluation.* A detailed physical examination was conducted preoperatively, at 1 year postoperatively, and usually at 2- to 3-year intervals thereafter until the latest follow-up visit. The last physical examination was conducted by 2 independent observers (AE and PY). The Harris hip score was recorded at each visit. At the last follow-up evaluation, patients were also asked to assess their satisfaction regarding the result.

*Radiographic evaluation.* Anteroposterior and frog-leg view (Lauenstein) radiographs were evaluated by an independent observer to determine loosening, radiolucencies, osteolysis, and heterotopic ossification (Brooker et al. 1973, Engh et al. 1987, Johnston et al. 1990). Polyethylene wear was assessed according to Charnley and Cupic (1973).

### Statistics

The Wilcoxon signed rank-sum test was used to compare pre-operative and postoperative Harris hip scores; when there were 2 measurements on 1 patient (bilateral cases), the mean value of these observations was used. The endpoint for survival was defined both as revision (removal or exchange of one component or the whole implant) for any reason and as revision because of aseptic loosening. Kaplan-Meier survival data were used to construct the survival probabilities of implants at 12 years, and the log-rank test was used to compare these probabilities. We considered p-values of < 0.05 to be significant. The statistical analyses were carried out using SPSS software version 14.0.

### Ethics

Informed consent was obtained from all participants. The authors obtained permission to perform this study from the ethics committee of the hospital district where the study was conducted (6.6.2003, dnro 278/E6/03).

## Results

### Intact hips

*Clinical results.* The Harris hip score for both intact and revised hips increased after the operation (p < 0.001 for all comparisons). Most of the intact hips and most of the revised hips were pain-free, and most of the patients were satisfied with the outcome (Table 4).

**Table 4.** Clinical results of intact and revised hips

Follow-up	HHS total **^a^**	HHS pain **^a^**	HHS > 80	Pain-free	Satisfied **^b^**
Intact hips (n = 26)					
Preoperatively	50 (14)	18 (12)	0	0	–
1-year	89 (8)	42 (9)	24 (92%)	21 (81%)	–
Final	92 (8)	43 (1)	24 (96%)	22 (92%)	21 (91%)
Revised hips (n = 35)					
Preoperatively	54 (19)	22 (13)	4 (11%)	0	–
1-year	85 (11)	42 (2)	24 (69%)	18 (51%)	–
Final	86 (14)	42 (6)	25 (71%)	24 (69%)	21 (66%)

**^a^** SD in parentheses.

**^b^** Satisfaction of patients.

Preoperatively, all patients had a limp, and the Trendelenburg sign was positive in 52/68 hips (Table 2). At the time of final follow-up, the abduction strength of the hip was graded as good or slightly reduced and the Trendelenburg sign was negative in 23 of 25 intact hips and in 30 of 35 revised hips.

The mean functional leg-length discrepancy decreased from 5 (1–10) cm before surgery to 2 (0–7) cm at the time of the final follow-up evaluation (p < 0.001).

26 hips in 23 patients retained both of the original components during the follow-up period. Of the 68 hips, 35 in 32 patients required revision during the follow-up period. 27 hips had 1 revision, 7 hips had 2 revisions, and 1 hip had 3 revisions.

### Reasons for failure

*Revisions for technical errors*. 2 stem revisions were caused by technical errors. In 1 patient, a CDH stem was inserted in excessive anteversion, leading to early dislocation. In a reoperation performed on the twelfth postoperative day, the malrotated stem was reinserted in the correct rotation. During insertion, one CDH stem perforated the posteromedial cortex of the femur. The malpositioning was evident in the postoperative radiographs, and a stem revision was performed on the eighth postoperative day. In 1 patient, a Vision cup was placed too medially. A revision operation was performed on the second postoperative day. A new cup was placed in a more lateral and cranial position, and autologous bone grafting was performed to augment the lower medial wall of the acetabulum. The revised cup was found to be radiographically well-fixed at the time of final follow-up.

*Revisions for implant errors.* 16 of the 18 threaded acetabular components were revised at a median of 3.7 years (2.1–13) postoperatively, and the remaining 2 were found to be loose radiographically at the time of final follow-up and were scheduled for cup revision. In 9 hips (7 patients: 2 Universal, 2 Mallory, and 3 Vision cups), the liner was revised due to excessive polyethylene wear. In each of these reoperations, a new liner (Biomet Vision) was cemented inside the well-fixed metallic shell. In addition, in 4 hips (4 patients: 2 Universal and 2 Mallory cups) the acetabular components were well fixed, but severe liner wear had led to wide granulomas. Thus, the cups had to be removed and the granulomas filled with allogeneous bone grafts, after which acetabular reconstruction was performed with antiprotrusion devices and cemented cups.

*Revisions for aseptic loosening.* Aseptic loosening was evident both clinically and radiographically in 1 press-fit, porous-coated cementless (Biomet Universal) acetabular component. It was revised with a larger Biomet Universal cup combined with autologous cancellous bone grafting due to a large bone defect in the medial wall of the acetabulum. 4 Biomet CDH stems were revised for loosening, at a median of 3.5 (3.3–5) years after the primary operation. All 4 stems were successfully revised with larger Biomet CDH stems. In all of these early revisions for stem loosening, a technical error caused the deficient primary stability and poor osseointegration. 2 of these 4 stems were clearly undersized, and in another 2 the greater trochanter had been detached so far distally that lateral support and rotational stability of the stem were deficient.

*Hips that were scheduled for revision at final follow-up.* At the time of final follow-up, 7 hips (in 6 patients) were scheduled for revision. 2 of these hips exhibited both migration of the socket and ≥ 2-mm wide radiolucent lines in at least 2 Gruen zones. These 2 patients were scheduled for revision of the threaded acetabular component. Furthermore, 4 hips in 3 patients were scheduled for liner revision due to excessive wear of the polyethylene liner, the depth of wear ranging from 1 to 3 mm. In addition, 1 hip with a 3-mm deep linear wear of the polyethylene liner also had a periacetabular osteolytic lesion that measured 450 mm^2^ and was in zone 3. The patient was scheduled for both liner revision and bone grafting of the osteolytic lesion.

*Radiographic results for intact hips.* Heterotopic ossification was observed in 2 of the 26 hips: Brooker class I in 1 hip and class III in 1 hip. Alignment of the femoral component was classified as neutral in all 26 hips. All cementless femoral components showed radiographic evidence of bone ingrowth at the last follow-up evaluation. Neither radiolucent lines nor femoral osteolysÍs were detected in any of these unrevised hips. Radiographic evidence of nonunion of the transposed trochanter was noted in 2 of the 68 hips (1 patient, bilateral nonunion).

The median acetabular cup angle was 48° (36–61). All bone grafts used to reinforce the acetabular roof united and the centralized medial walls of the acetabula healed without complications. At the time of the final follow-up evaluation, 2 of the 26 intact hips showed linear wear of the polyethylene liner, the depth of wear ranging from 0.5 to 1 mm.

**Figure 3.**
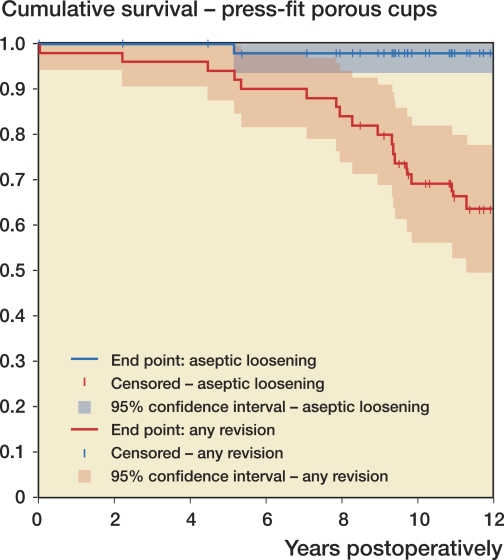
Kaplan-Meier survival curve for the press-fit porous-coated Figure 4. Kaplan-Meier survival curve for the CDH femoral compo-acetabular components, with cup revision because of aseptic loosennents, with stem revision for any reason as the endpoint. CI: confi-ing and cup revision for any reason as the endpoints. CI: confidence dence interval. interval.

**Figure 4.**
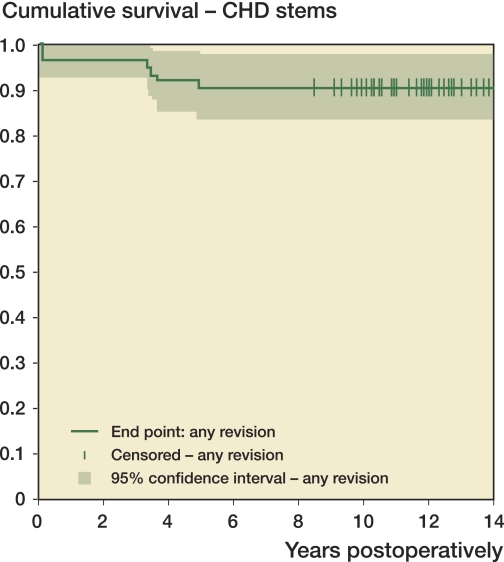
Kaplan-Meier survival curve for the CDH femoral components, with stem revision for any reason as the endpoint. CI: confi-dence interval.

*Survivorship analysis.* The 12-year survival rate for the press-fit porous-coated acetabular components (Biomet Universal, Biomet Mallory, and Biomet Vision) was 98% (95% confidence interval (CI): 94–100) with revision because of aseptic loosening as the endpoint, and 64% (CI: 50–78) with revision of the cup for any reason as the endpoint (Figure 3). The overall survival rate for press-fit, porous-coated cups was better than that for smooth-threaded cups (p < 0.001). The survival rate for the Biomet CDH stem was 93% (CI: 87–100) at 12 years with revision for aseptic loosening as the endpoint and 88% (CI: 81–96) when the endpoint was defined as stem revision for any reason (Figure 4).

*Complications.* There were 12 perioperative complications: 2 instances of peroneal nerve palsy, 2 femoral nerve palsies (1 resolved), 1 superior gluteal nerve palsy (resolved), 1 obturatorius nerve palsy (resolved), 5 non-displaced fractures of the proximal part of the femur, and 1 superficial wound infection (Table 5).

**Table 5.** Complications

Complication	n	Etiology	Treatment	Outcome
Peroneal nerve palsy #1	2	Hematoma	Surgical evacuation on the 1st POD	Permanent incomplete dropfoot
Peroneal nerve palsy #2		Too much offset, ischial nerve impinging against acetabulum	Exchange of femoral head to a shorter one on the 2nd POD	Permanent incomplete dropfoot
Femoral nerve palsy #1	2	Hematoma	Surgical evacuation on the 5th POD	Spontaneous recovery in 3 months
Femoral nerve palsy #2		Hematoma	Surgical evacuation on the 1st POD	Motor paresis fully resolved in 6 months, permanent sensory deificit in thigh
Superior gluteal nerve plasy	1	Perioperative neuropraxia	Watchful waiting	Spontaneous recovery in 6 months
Obturatorius nerve palsy	1	Perioperative neuropraxia	Watchful waiting	Spontaneous recovery in 6 months
Non-displaced fracture of	5	1 identified during rasping,	3 fixed with Parham bands and	All united without reoperations
proximal femur		4 during stem insertion	2 fixed with Dall-Miles cables perioperatively	
Superficial wound infection	1	*Staphylococcus aureus*	Peroral antibiotics	Fully resolved

POD: postoperative day.

## Discussion

We found that cementless total hip arthroplasty with femoral shortening osteotomy substantially reduced pain and improved hip joint function in adult patients with a previous Schanz osteotomy of the femur. However, complications occurred more frequently than after ordinary total hip arthroplasty and the rate of revisions related to poor acetabular components was high. However, with the techniques we used and the prosthetic components available nowadays, a good long-term clinical outcome can be expected.

The choice between the two femoral shortening osteotomy methods is always made before surgery. We recommend the use of femoral shortening osteotomy combined with distal advancement of the greater trochanter in all suitable cases because, in our experience, it gives better abductor strength and better motion than the segmental shortening technique despite the more anatomical radiographic result achieved with the latter technique. We recommend that segmental subtrochanteric shortening osteotomy should be used only in hips in which the Schanz osteotomy has been performed so low that the former technique would result in inadequate limb-length correction. In such hips, we currently stabilize the subtrochanteric osteotomy with a distally fluted modular femoral component, which both facilitates and accelerates the procedure, as rotational stability can be achieved without the step method. The younger the patient, the more we try to achieve equal limb length. Various sizes of lifts must be carefully tested preoperatively for patients with fixed degenerative changes in the lower back to determine the desired functional outcome (Hoikka et al. 1993).

The cup should always be seated at the anatomical level (Yoder et al. 1988, Karachalios et al. 1993, Sener et al. 2002, Carlsson et al. 2003, Hartofilakidis and Karachalios 2004, Perka et al. 2004), or even lower if the anteroposterior diameter of the pelvic bone is too small at the anatomical level. Clinically, a high acetabulum perpetuates abductor insufficiency, limping, and limb-length discrepancy.

In our series, all threaded cementless cups loosened, which confirms previous reports (Engh et al. 1990, Tallroth et al. 1993). In addition, excessive polyethylene wear of the modular cementless cups used resulted in numerous revisions—a commonly reported problem with these cup designs (Puolakka et al. 2001). Today, we prefer cementless press-fit porous-coated cups with modern hard-on-hard articulations for these patients.

There were 6 nerve palsies in our series, 4 of which resolved completely. To avoid nerve injury, we use intraoperative electroneuromyography when we attempt to increase limb length more than 3 cm. In addition, postoperatively, we position the extremity with the hip fully extended and the knee flexed to reduce tension on the sciatic nerve (Morscher 1995).

Preparation of the hard cortical femur was difficult and resulted in five fractures during rasping or insertion of the pros-thesis. In spite of our protocol of early mobilization, dislocations were very rare in our series. Nevertheless, the complication rate is comparable to that reported in other studies of total hip arthroplasty for severely dysplastic joints (Paavilainen et al. 1993, Carlsson et al. 2003, Hartofilakidis and Karachalios 2004, Perka et al. 2004).

In summary, cementless total hip arthroplasty with placement of the cup at the level of the true acetabulum and femoral shortening osteotomy combined with distal advancement of the greater trochanter can be recommended for most patients with a previous Schanz osteotomy of the femur, irrespective of the etiology of the hip disease. For patients with a low-seated unilateral Schanz osteotomy, we prefer subtrochanteric segmental resection and angular correction because it gives better correction of leg length.
